# Association of Allostatic Load With All-Cause Mortality in Patients With Breast Cancer

**DOI:** 10.1001/jamanetworkopen.2023.13989

**Published:** 2023-05-18

**Authors:** Samilia Obeng-Gyasi, Mohamed I. Elsaid, Yurong Lu, JC Chen, William E. Carson, Tarah J. Ballinger, Barbara L. Andersen

**Affiliations:** 1 Division of Surgical Oncology, Department of Surgery, The Ohio State University, Columbus; 2 Department of Biomedical Informatics, College of Medicine, The Ohio State University, Columbus; 3 Secondary Data Core, Center for Biostatistics, College of Medicine, The Ohio State University, Columbus; 4 Department of Medicine, Indiana University School of Medicine, Indianapolis; 5 Department of Psychology, The Ohio State University, Columbus

## Abstract

**Importance:**

Elevated allostatic load (AL) has been associated with adverse socioenvironmental stressors and tumor characteristics that convey poor prognosis in patients with breast cancer. Currently, the association between AL and all-cause mortality in patients with breast cancer is unknown.

**Objective:**

To examine the association between AL and all-cause mortality in patients with breast cancer.

**Design, Setting, and Participants:**

This cohort study used data from an institutional electronic medical record and cancer registry at the National Cancer Institute Comprehensive Cancer Center. Participants were patients with breast cancer diagnoses (stage I-III) between January 1, 2012, through December 31, 2020. Data were analyzed from April 2022 through November 2022.

**Exposure:**

AL was expressed as a summary score calculated by assigning 1 point for biomarkers in the worst sample quartile. High AL was defined as AL greater than the median.

**Main Outcomes and Measures:**

The main outcome was all-cause mortality. A Cox proportional hazard models with robust variance tested the association between AL and all-cause mortality.

**Results:**

There were 4459 patients (median [IQR] age, 59 [49-67] years) with an ethnoracial distribution of 3 Hispanic Black patients (0.1%), 381 non-Hispanic Black patients (8.5%), 23 Hispanic White patients (0.5%), 3861 non-Hispanic White patients (86.6%), 27 Hispanic patients with other race (0.6%), and 164 non-Hispanic patients with other race (3.7%). The mean (SD) AL was 2.6 (1.7). Black patients (adjusted relative ratio [aRR], those with 1.11; 95% CI, 1.04-1.18), single marital status (aRR, 1.06; 95% CI, 1.00-1.12), and those with government-supplied insured (Medicaid aRR, 1.14; 95% CI, 1.07-1.21; Medicare aRR, 1.11; 95% CI, 1.03-1.19) had a higher adjusted mean AL than those who were White, married/living as married, or privately insured, respectively. Adjusting for sociodemographic, clinical, and treatment factors, high AL was associated with a 46% increase in mortality risk (hazard ratio [HR], 1.46; 95% CI, 1.11-1.93) over low AL. Similarly, compared with patients in the first AL quartile, those in the third quartile (HR, 1.53; 95% CI, 1.07-2.18) and the fourth quartile (HR, 1.79; 95% CI, 1.16-2.75) had significantly increased risks of mortality. There was a significant dose-dependent association between increased AL and a higher risk of all-cause mortality. Furthermore, AL remained significantly associated with higher all-cause mortality after adjusting for the Charlson Comorbidity Index.

**Conclusions and Relevance:**

These findings suggest increased AL is reflective of socioeconomic marginalization and associated with all-cause mortality in patients with breast cancer.

## Key Points

**Question:**

Is there an association between allostatic load and all-cause mortality in patients with breast cancer?

**Findings:**

In this cohort study of 4459 patients with stage I to III breast cancer, increased allostatic load was associated with a higher risk of all-cause mortality after adjusting for the Charlson Comorbidity Index and sociodemographic, clinical, and treatment factors.

**Meaning:**

These findings suggest that in patients with nonmetastatic breast cancer, allostatic load is associated with all-cause mortality.

## Teaser

This cohort study evaluates the association of allostatic load with all-cause mortality in patients with breast cancer.

## Introduction

Allostatic load (AL) is a cumulative measure of physiologic damage secondary to cognitive-emotional responses and perceptions to socioenvironmental stressors (ie, low socioeconomic status).^1^ McEwen and Stellar’s seminal article^2^ on AL defines it as the “cost of chronic exposure to fluctuating or heightened neural or neuroendocrine response resulting from repeated or chronic environmental challenge(s).” AL is measured by combining primary mediators (eg, cortisol) of the hypothalamic-pituitary-adrenal axis and the sympathetic adrenal medullary pathway, secondary outcomes of the hypothalamic-pituitary-adrenal axis and sympathetic adrenal medullary pathway (eg, c-reactive protein), and tertiary outcomes (eg, cancer) into a composite score.^3,4,5^ Although no reference standard of biomarkers are used to calculate AL, most studies use a combination of secondary and tertiary outcomes.^6,7^ Emerging literature suggests elevated AL (ie, an indicator of worsened physiologic dysregulation), is associated with exposure to adverse socioenvironmental stressors (eg, low socioeconomic status, membership in marginalized and minoritized groups),^8^ an increased risk of developing chronic diseases such as cancer,^9^ and worse all-cause mortality.^10^

In patients with cancer, elevated AL has been associated with worse all-cause and disease-specific mortality.^11,12^ For example, in our study examining AL in patients with metastatic lung cancer, an elevated AL at diagnosis was associated with a 43% higher all-cause mortality.^13^ Additionally, elevated AL was associated with negative stressors such as limited mobility, worse self-care, problems engaging with usual activities, and a high number of stressful life events.^13^ Among patients with breast cancer, high AL compared with low AL has been associated with larger tumor size and estrogen receptor–negative tumors.^14,15^ Furthermore, patients with breast cancer reporting marital dissolution, low educational attainment, and engagement in unhealthy behaviors (eg, low exercise or smoking) have a higher AL than those who are married, have higher educational attainment, and participate in healthy lifestyle behaviors.^14^

To date, there are no studies focusing exclusively on the association between AL and all-cause mortality in patients with breast cancer.^16^ The objective of this cohort study is to (1) examine differences in sociodemographic, clinical, and treatment characteristics between patients with high vs low AL and (2) evaluate the association between AL and all-cause mortality in patients with breast cancer.

## Methods

### Data Source

The Ohio State University Cancer Registry and electronic medical record (IHIS) were queried for patients with stages I to III breast cancer, aged 18 years or older, who received surgical treatment (mastectomy or lumpectomy) at the Ohio State University Comprehensive Cancer Center from January 1, 2012, through December 31, 2020. Surgical management was an inclusion criterion as (1) most of the biomarkers used to calculate AL are routinely collected as part of the preoperative workup^7^ and (2) most patients with stages I to III breast cancer undergo surgical management.^17^ Patients who did not receive surgical treatment, those with initial diagnoses of stage 0 or IV breast cancer, and those with unknown breast cancer molecular subtypes were excluded (Figure). Ten imputation data sets were created to address missing values. The Strengthening the Reporting of Observational Studies in Epidemiology (STROBE) reporting guidelines were followed in the design, analysis, and interpretation of study results. The Ohio State University Office of Responsible Research Practices approved this study. Informed consent was waived by the institutional review board since this is a retrospective review.

**Figure.**
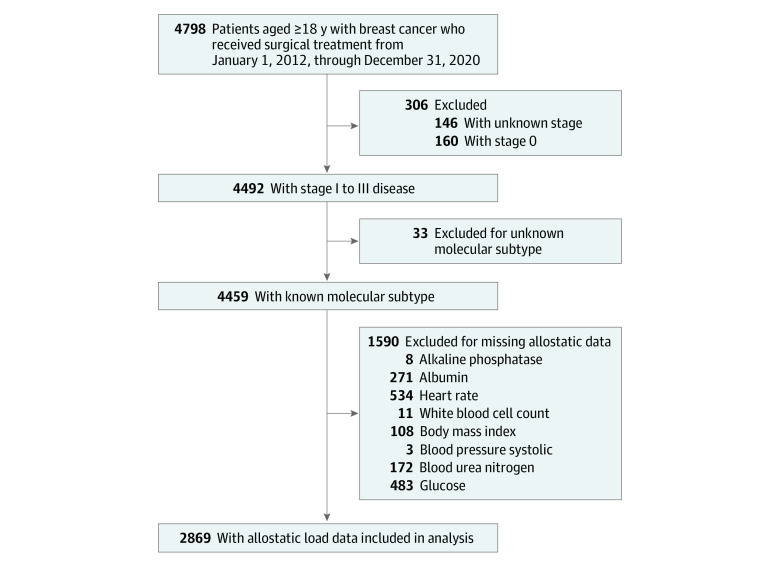
Study Schema

### Study Measures

#### Allostatic Load

There is currently no reference standard for which biomarkers to include in the calculation of allostatic load (AL). Consequently, for this study, biomarkers routinely collected in clinical practice and frequently used in AL literature were used.^7^ Biomarkers for AL were retrieved from IHIS and included if they were collected up to 12 months before or 6 months after biopsy-proven breast cancer diagnosis. The AL measure was derived from 4 physiologic systems (1) cardiovascular—heart rate, blood pressure (systolic and diastolic); (2) metabolic—body mass index (BMI), alkaline phosphatase, blood glucose, and albumin; (3) renal—creatinine, blood urea nitrogen; and (4) immune—white blood cell count.

AL was calculated using the quartile method. This method is the most common method of calculating AL in the literature.^18^ The distribution of each biomarker in the study cohort was ascertained.^6^ Patients were assigned a point if the biomarker was in the worst quartile for that biomarker. Specifically, patients with heart rate, blood pressure (systolic and diastolic), alkaline phosphatase, blood glucose, BMI, creatinine, blood urea nitrogen, and white blood cell counts in the 75th percentile or higher were awarded a point. Conversely, albumin in the 25th percentile or lower was awarded a point. All assigned points were combined into a composite AL summary score with a range between 0 and 10. Higher scores are indicative of worse physiologic dysregulation. AL was dichotomized into high vs low, with high AL defined as a total AL score greater than the median. We also grouped the composite AL summary score into quartiles.

#### Sociodemographic Factors

Sociodemographic information included age, self-reported race, self-reported ethnicity, marital status (single, married/living as married, widowed, separated, or divorced), insurance (private, Medicaid, Medicare, or other), smoking history (never, current, or former smoker) and alcohol use (never, current, or former). Racial categories were Black, White, and Other. Due to their small sample sizes, Asian, American Indian, Alaskan Native, Native Hawaiian, Other Pacific Islander, and multiracial individuals were collapsed into the other category. Ethnicity was dichotomized as Hispanic or non-Hispanic. Race and ethnicity in this study are social constructs and not reflective of genetic ancestry.^19^ Furthermore, race was examined to ascertain differences in AL by racial groups. Comorbidity burden was calculated using the National Cancer Institute (NCI) modified Charlson Comorbidity Index,^20^ which excludes cancer.

#### Clinical and Treatment Characteristics

Clinical characteristics included stage, hormone (estrogen, progesterone) receptor (HR) status, and human epidermal growth factor receptor 2 (ERBB2) receptor status. Patients were classified according to the Surveillance, Epidemiology, and End Result (SEER) program breast cancer subtypes (HR positive/ERBB2 negative, HR negative/ERBB2 negative, HR negative/ERBB2 positive, and HR positive/ERBB2 positive).^21^ Surgical treatments included breast surgery (mastectomy, lumpectomy) and axillary surgery (sentinel lymph node biopsy, axillary lymph node dissection). Systemic treatments (chemotherapy, hormone therapy) and radiation therapy were also included. Surgical complications were dichotomized as yes or no (eTable 1 in Supplement 1).

### Outcomes

#### Primary Outcome

The primary outcome was the duration from the date of breast cancer diagnosis to the date of death. For participants presumed to be alive, follow-up was calculated from the date of cancer diagnosis to the date of loss to follow-up or the end of the study (December 31, 2020), whichever came first.

### Statistical Analysis

To address missing data, all missing values were imputed using multiple imputations by chained equations to create 10 imputed data sets.^22^ The imputation by chained equations approach uses a flexible variable-by-variable multivariable imputation model to address missing data for data sets with complex data structures. As such, we used logistic regression-based imputation models to impute binary and ordinal variables, the discriminant function to impute nominal variables, and a regression-based approach with projected mean matching to impute continuous variables. All imputation-corrected parameters and SEs were combined using the Rubin method.^23^

Overall and stratified characteristics were summarized using descriptive statistics, including means and standard deviations (SD) for continuous variables and frequencies and proportions for categorical variables. Differences between patients with low vs high AL were compared using the Mann-Whitney U test for continuous variables and χ^2^ or Fisher exact tests for categorical variables. The associations between the mean AL sum score and sociodemographic factors were examined using analysis of variance (ANOVA). We used modified Poisson regression models to examine the adjusted associations, on the relative ratios (RR) scale, between sociodemographic factors and the AL sum score.^24^ Incidence mortality rates by (1) high AL status and (2) AL-quartile were calculated using the number of deaths divided by 100 person-years of follow-up.

Crude and fully adjusted Cox proportional hazard models with robust variance were used to test the outcome of AL on the risk of all-cause mortality. The proportional hazard assumption was tested by adding a time-dependent function of AL status to the regression models. The dose-response associations between the AL sum score and mortality risk were evaluated using a 3-knot restricted cubic spline in the adjusted Cox proportional hazard models. The 3 knots were placed at the 10th, 50th, and 90th percentile of the AL sum score.^25^ Wald-χ^2^ tests were used to assess the overall and nonlinear associations between the AL score percentiles and mortality risk.

A secondary analysis examined the association between all-cause mortality and each AL biomarker using established clinical cut-offs.^13^ As such, univariate Cox proportional hazard models were fitted with each AL biomarker as the exposure to discern its association with mortality risk. Furthermore, an adjusted Cox proportional hazard model that included all AL biomarkers and high AL status was fitted to examine the utility of AL as an independent forecast of all-cause mortality in breast cancer. In a sensitivity analysis, we further adjusted our dose-response models to include the Charlson Comorbidity Index to assess the robustness of our main findings. A 2-sided *P* value of less than .05 was considered statistically significant. All analyses were performed using SAS 9.4 software (SAS Institute). Data were analyzed from April 2022 to November 2022.

## Results

### Description of Study Population

After multiple imputation of patients with missing values who were identified during the study period, 4459 patients in the Ohio State University Cancer Registry and IHIS met study criteria (Figure). The proportion of patients with missing clinical cancer stage and molecular subtype was 7.0%, while 33% of all patients had a missing value for at least 1 of the AL biomarkers. The median (IQR) age was 59 (49-67) years. Patients had an ethnoracial distribution of 3 Hispanic Black patients (0.1%), 381 non-Hispanic Black patients (8.5%), 23 Hispanic White patients (0.5%), 3861 non-Hispanic White patients (86.6%), 27 Hispanic patients with other race (0.6%), and 164 non-Hispanic patients with other race (3.7%). Overall, most patients were married (2834 patients [63.8%]), privately insured (2650 patients [59.4%]), and had stage I disease (2814 patients [63.1%]), HR-positive/ERBB2-negative breast cancers (2753 patients [61.7%]), and no comorbidities (3520 patients [78.9%]). Biological sex of the sample was 100% female. Almost half of the patients underwent mastectomy (2124 patients [47.6%]), and 365 (8.2%) had postoperative complications (Table 1). The mean (SD) AL score for the study sample was 2.6 (1.7) with a median (IQR) of 2.0 (1.0-4.0) (eTable 2 in Supplement 1).

**Table 1.** Overview of Sociodemographic, Clinical and Treatment Characteristics by Low vs High Allostatic Load^a^

Patient characteristics	All (N = 4459)	Low allostatic load (n = 2257)	High allostatic load (n = 2202)	*P* value^b^
Age, y				
Mean (SD)	58.2 (12.5)	56.3 (12.7)	60.2 (12.8)	<.001
Median (IQR)	59.0 (49.0-67.0)	56.0 (47.0-65.0)	60.0 (51.0-68.0)	
Age group, No. (%)				
≤39, y	313 (7.0)	195 (8.6)	118 (5.4)	<.001
40-49, y	838 (18.8)	496 (22.0)	343 (15.6)	
50-59, y	1184 (26.6)	624 (27.6)	560 (25.4)	
60-59, y	1286 (28.8)	609 (27.0)	677 (30.7)	
≥70, y	838 (18.8)	333 (14.8)	505 (22.9)	
Race and ethnicity, No. (%)				
Hispanic Black	3 (0.1)	3 (0.1)	0 (0.0)	<.001
Non-Hispanic Black	381 (8.5)	149 (6.6)	232 (10.5)	
Hispanic White	23 (0.5)	8 (0.4)	15 (0.7)	
Non-Hispanic White	3861 (86.6)	1972 (87.4)	1889 (85.8)	
Hispanic other^c^	27 (0.6)	19 (0.8)	8 (0.4)	
Non-Hispanic other^c^	164 (3.7)	106 (4.7)	58 (2.6)	
Marital status, No. (%)				
Single	639 (14.3)	309 (13.7)	330 (15.0)	<.001
Married/living as married	2843 (63.8)	1539 (68.2)	1304 (59.2)	
Widowed, separated or divorced	977 (21.9)	409 (18.1)	568 (25.8)	
Health insurance, No. (%)				
Private	2650 (59.4)	1497 (66.3)	1153 (52.4)	<.001
Medicaid	1365 (30.6)	552 (24.5)	813 (36.9)	
Medicare	376 (8.4)	172 (7.6)	204 (9.3)	
Other	68 (1.5)	37 (1.6)	32 (1.5)	
Smoking history, No. (%)				
Never	2787 (62.5)	1442 (63.9)	1345 (61.1)	.124
Current or former	1672 (37.5)	815 (36.1)	858 (39.0)	
Alcohol use, No. (%)				
Never	2103 (47.2)	929 (41.2)	1174 (53.3)	<.001
Current or former	2356 (52.8)	1328 (58.8)	1028 (46.7)	
ERBB2 receptor status, No. (%)				
Negative	3767 (84.5)	1945 (86.2)	1822 (82.7)	.002
Positive	692 (15.5)	312 (13.8)	380 (17.3)	
Progesterone receptor status, No. (%)				
Negative	1348 (30.2)	668 (29.6)	680 (30.9)	.40
Positive	3111 (69.8)	1589 (70.4)	1522 (69.1)	
Estrogen receptor status, No. (%)				
Negative	890 (20.0)	423 (18.7)	467 (21.2)	.05
Positive	3569 (80.0)	1834 (81.3)	1735 (78.8)	
Molecular subtype, No. (%)				
HR negative/ERBB2 positive	242 (5.4)	120 (5.3)	122 (5.5)	.007
HR positive/ERBB2 negative	2753 (61.7)	1438 (63.7)	1315 (59.7)	
HR positive/ERBB2 positive	818 (18.3)	398 (17.6)	420 (19.1)	
HR negative/ERBB2 negative	646 (14.5)	301 (13.3)	345 (15.7)	
Cancer stage, No. (%)				
1	2814 (63.1)	1494 (66.2)	1320 (59.9)	<.001
2	1369 (30.7)	644 (28.5)	725 (32.9)	
3	276 (6.2)	119 (5.3)	157 (7.1)	
Mastectomy, No. (%)	2124 (47.6)	1109 (49.1)	1015 (46.1)	.06
Lumpectomy, No. (%)	2306 (51.7)	1134 (50.2)	1172 (53.2)	.05
Sentinel lymph node biopsy only, No. (%)	1444 (32.4)	751 (33.3)	693 (31.5)	.22
Axillary lymph node biopsy only, No. (%)	237 (5.3)	110 (4.9)	127 (5.8)	.23
Both sentinel and axillary lymph node biopsies, No. (%)	2013 (45.1)	1046 (46.3)	967 (43.9)	.13
Surgical complications, No. (%)	365 (8.2)	152 (6.7)	213 (9.7)	.001
Hormone therapy, No. (%)	3355 (75.2)	1736 (76.9)	1619 (73.5)	.01
Radiation therapy, No. (%)	2679 (60.1)	1331 (59.0)	1348 (61.2)	.15
Chemotherapy, No. (%)	2112 (47.4)	1031 (45.7)	1081 (49.1)	.03
Charlson Comorbidity Index, No. (%)^a^				
0	3520 (78.9)	1936 (85.8)	1584 (71.9)	<.001
1-3	826 (18.5)	296 (13.1)	530 (24.1)	
≥4	113 (2.5)	25 (1.1)	89 (4.0)	

Abbreviations: ERBB2, Human epidermal growth factor receptor 2; HR, hormone receptor.

^a^ Using Charlson Index weights (excluding cancer).

^b^ *P* values from χ^2^ tests for the association between allostatic load and patient characteristics, *P* value for age Wilcoxon rank sum test.

^c^ Other racial categories include Asians, American Indians, Alaskan Natives, Native Hawaiian, Other Pacific Islanders, and multiracial individuals.

Compared with patients in the low AL group, patients in the high AL group were older (median [IQR] 60 [51-68] years vs 56 (47-65) years, unpartnered (single, 330 patients [15.0%] vs 309 patients [13.7%], widowed/separated or divorced 568 patients [25.8%] vs 409 patients [18.1%]), and government insured (Medicaid 813 patients [36.9%] vs 552 patients [24.5%], Medicare 204 patients [9.3%] vs 127 patients [7.6%]). A higher proportion of individuals in the high AL group identified as non-Hispanic Black race (232 patients [10.5%]) than those in the low AL group (149 patients [6.6%]). Patients in the high AL group were more likely to have 1 or fewer comorbidities than those in the low AL group (1-3 comorbidities, 530 patients [24.1%] vs 296 patients [13.1%]; ≥4 comorbidities, 89 patients [4.0%] vs 25 patients [1.1%]). Notably, a slightly higher percentage of patients with a high AL presented with HR negative/ERBB2 negative breast cancer (345 patients [15.7%] vs 301 patients [13.3%]) or HR positive/ERBB2 negative cancer (420 patients [19.1%] vs 398 patients [17.6%]) than patients with low AL. There were no significant differences between the groups on breast or axillary surgical management (Table 1). However, more patients in the high AL group experienced postoperative complications (213 patients [9.7%] vs 152 patients [6.7%]).

The age-adjusted mean AL score was higher for patients with Black race (RR, 3.08; 95% CI, 2.91-2.35), single marital status (RR, 2.76; 95% CI, 2.62-2.89), widowed, separated, or divorced marital status (RR, 2.78; 95% CI, 2.66-2.90), and government insurance (Medicaid RR, 2.82; 95% CI, 2.70-2.95; Medicare RR, 2.90; 95% CI, 2.73-3.08) than those who were White, married or living as married, or privately insured (Table 2). The fully adjusted mean AL score was 11% higher for Black vs White patients (aRR, 1.11; 95% CI, 1.04-1.18). Compared with married or living as married patients, the adjusted mean AL score was 6% higher for single patients (aRR, 1.06; 95% CI, 1.00-1.12) and 8% higher for widowed, separated, or divorced marital status (aRR, 1.08; 95% CI, 1.03-1.13). Patients with Medicaid and Medicare insurance had higher mean AL scores by 14% and 11%, respectively, compared with those with private insurance (Medicaid aRR, 1.14; 95% CI, 1.07-1.21; Medicare aRR, 1.11; 95% CI, 1.03-1.19).

**Table 2.** Crude and Adjusted Associations Between Allostatic Load Aggregate Score and Sociodemographic Factors

Variable	Allostatitc load aggregate score, mean (95% CI)^a^		Adjusted relative ratio (95% CI)^b^
	Crude	Age-adjusted	
**Allostatic Load Aggregate Score**			
Race			
White^c^	2.61 (2.56-2.66)	2.53 (2.47-2.59)	1 [Reference]
Black	3.13 (2.95-3.30)	3.08 (2.91-3.25)	1.11 (1.04-1.18)
Other	2.02 (1.78-2.27)	2.05 (1.81-2.30)	0.79 (0.70-0.89)
Ethnicity			
Non-Hispanic^c^	2.63 (2.58-2.68)	2.56 (2.50-2.61)	1 [Reference]
Hispanic	2.38 (1.91-2.85)	2.44 (1.98-2.90)	1.05 (0.70-0.89)
Marital status			
Married/living as married^c^	2.49 (2.42-2.55)	2.43 (2.36-2.50)	1 [Reference]
Single	2.73 (2.60-2.87)	2.76 (2.62-2.89)	1.06 (1.00-1.12)
Widowed, separated or divorced	2.97 (2.86-3.09)	2.78 (2.66-2.90)	1.08 (1.03-1.13)
Health insurance			
Private^c^	2.37 (2.31-2.44)	2.37 (2.29-2.44)	1 [Reference]
Medicaid	3.08 (2.99-3.17)	2.82 (2.70-2.95)	1.14 (1.07-1.21)
Medicare	2.83 (2.66-3.00)	2.90 (2.73-3.08)	1.11 (1.03-1.19)
Other	2.55 (2.12-2.97)	2.51 (2.08-2.93)	1.03 (0.86-1.22)

^a^ Poission regression with robust error variance adjusted for age group, history of alcohol consumption, ever-smoker, molecular subtype, cancer stage, mastectomy, lumpectomy, surgical complications, hormone therapy, radiation therapy, chemotherapy, sentinel lymph node biopsy only, axillary lymph node biopsy only, both sentinel and axillary lymph node biopsies.

^b^ Allostatic load included biomarkers for alkaline phosphatase, albumin, creatinine serum, heart rate, white blood cell count, body mass index (BMI), blood pressure diastolic, blood pressure systolic, blood urea nitrogen, and glucose; total score range (0 to 10).

^c^ Reference group.

### AL and All-Cause Mortality

After adjusting for sociodemographic, clinical, and treatment factors, high AL was significantly associated with a higher risk of all-cause mortality than low AL (hazard ratio [HR], 1.46; 95% CI, 1.11-1.93) (Table 3). Furthermore, when AL was examined as quartiles, compared with patients in the lowest quartile (Q1), those in the highest 2 quartiles (Q3: HR, 1.53; 95% CI, 1.07-2.18; Q4: HR, 1.79; 95% CI, 1.16-2.75) had worse all-cause mortality. Increases in AL were associated with a higher risk of all-cause mortality in all dose-response analyses (eFigure 1 in Supplement 1). In adjusted dose-response analyses, an increase in AL score was associated with worsening all-cause mortality (Table 4). High AL remained significantly associated with increased risk of all-cause mortality when further adjusted for the Charlson Comorbidity Index (eFigure 2 in Supplement 1). In secondary adjusted analysis, including all biomarkers and high AL status, high AL remained significantly associated with higher risk of all-cause mortality (high AL vs low AL HR, 1.51; 95% CI, 1.11-2.07) (eTable 3 in Supplement 1).

**Table 3.** Association Between Allostatic Load and All-Cause Mortality in Patients with Breast Cancer

Allostatic load^a^	Participants, No.	Events, No.	Person-years	Mortality rate per 100 person-years (95% CI)	Absolute rate difference per 100 person-years (95% CI)	HR (95% CI)	
						Crude	Adjusted^b^
AL							
Low	2257	99	9541.3	1.04 (0.84 to 1.26)	[Reference]	1 [Reference]	1 [Reference]
High	2202	180	9026.3	1.99 (1.71 to 2.31)	0.96 (0.60 to 1.31)	1.93 (1.50 to 2.50)	1.46 (1.11 to 1.93)
AL-quartile groups							
Q1	1255	48	5369.0	0.89 (0.66 to 1.19)	1 [Reference]	1 [Reference]	1 [Reference]
Q2	1002	51	4172.3	1.22 (0.91 to 0.16)	0.33 (−0.92 to 7.48)	1.35 (0.88 to 2.07)	1.15 (0.74 to 1.81)
Q3	1576	118	6635.1	1.78 (1.47 to 2.13)	0.88 (4.76 to 1.29)	1.99 (1.41 to 2.81)	1.53 (1.07 to 2.18)
Q4	626	62	2391.3	2.59 (1.99 to 3.32)	1.70 (1.01 to 2.39)	2.93 (1.97 to 4.34)	1.79 (1.16 to 2.75)

Abbreviations: AL, allostatic load; HR, hazard ratio.

^a^ Allostatic load included biomarkers for alkaline phosphatase, albumin, creatinine serum, heart rate, white blood cell count, body mass index (BMI), blood pressure diastolic, blood pressure systolic, blood urea nitrogen, and glucose. High allostatic load was defined as a total allostatic load score (range 0 to 10) greater than the median.

^b^ Models were adjusted for age group, race, ethnicity, health insurance, marital status, history of alcohol consumption, ever-smoker, molecular subtype, cancer stage, mastectomy, lumpectomy, surgical complications, hormone therapy, radiation therapy, chemotherapy, sentinel lymph node biopsy only, axillary lymph node biopsy only, both sentinel and axillary lymph node biopsies.

**Table 4.** Crude and Adjusted HRs of All-Cause Mortality Per 1 Unit Increase in Allostatic Load Scores Relative to 0 Allostatic Load Score

Allostatic Load^a^	HR (95% CI)	
	Crude	Adjusted^b^
0	1 [Reference]	1 [Reference]
1	1.41 (1.14-1.73)	1.25 (1.01-1.54)
2	1.94 (1.32-2.87)	1.53 (1.03-2.28)
3	2.48 (1.55-3.97)	1.77 (1.09-2.85)
4	2.96 (1.85-4.73)	1.93 (1.19-3.12)
5	3.40 (2.16-5.34)	2.05 (1.28-3.29)
6	3.89 (2.46-6.16)	2.18 (1.33-3.56)
7	4.45 (2.71-7.32)	2.31 (1.33-3.99)
8	5.09 (2.91-8.91)	2.45 (1.30-4.59)
9	5.83 (3.07-11.05)	2.59 (1.25-5.37)
10	6.67 (3.21-13.85)	2.75 (1.19-6.36)

Abbreviation: HR, hazard ratio.

^a^ The allostatic load score was modeled using a 3-knot restricted cubic spline.

^b^ Models were adjusted for age group, race, ethnicity, health insurance, marital status, history of alcohol consumption, ever-smoker, molecular subtype, cancer stage, mastectomy, lumpectomy, surgical complications, hormone therapy, radiation therapy, chemotherapy, sentinel lymph node biopsy only, axillary lymph node biopsy only, both sentinel and axillary lymph node biopsies.

## Discussion

This large retrospective analysis of patients receiving surgical management for early-stage breast cancer at a single comprehensive cancer center found that elevated AL is associated with higher all-cause mortality. Furthermore, Black race, unpartnered marital status, and government insurance were associated with a higher AL than White race, being partnered, or having private insurance, respectively. These results support existing studies suggesting patients experiencing persistent socioeconomic marginalization (eg, Black people or Medicaid insured) have higher biological correlates of stress, operationalized as AL, than their socioeconomically privileged counterparts (eg, White people or private insurance).

The association between elevated AL and worse all-cause mortality is consistent with prior studies.^26^ Specifically, a recent systematic review and meta-analysis by Parker et al^10^ showed high AL is associated with a 22% increase in the risk of all-cause mortality in patients with and without personal histories of cancer. Our current finding of a 46% or higher increase in all-cause mortality, with AL dichotomized (low vs high) or as quartiles, is consistent with our prior findings^13^ in the metastatic lung cancer cohort of HR 1.43 (95% CI, 1.16-1.79). Differences in the risk of all-cause mortality across studies are most likely attributable to differences in the study populations, AL biomarkers, and how AL was calculated. For instance, to ensure the reproducibility of our AL measure in clinical practice, only biomarkers routinely collected as part of the preoperative workup were included in the AL measure for this study. Furthermore, in conjunction with our lung cancer study, this study is one of few to have included treatment variables (locoregional and systemic treatments) as confounding variables in models examining the association between AL and all-cause mortality.

The association of AL with all-cause mortality was similar across varied permutations of AL (eg, continuous, dichotomized, and quartiles), illustrating the consistent association between our AL measure and all-cause mortality. The pathways of how AL exerts its effect on all-cause mortality is an area of active research. Our results support the bifactor model suggested by Wiley et al,^27^ which proposes AL biomarkers independently affect clinical outcomes while concomitantly functioning through allostatic load as a common factor. This concept is similarly illustrated in our study, given the association of multiple comorbidities with a high AL and the persistent association between increased AL and worse all-cause mortality after controlling for the Charlson Comorbidity Index. Additionally, although individual biomarkers were associated with all-cause mortality, the association between increased AL and all-cause mortality persisted after adjusting for the biomarkers.

This study shows that vital signs (eg, blood pressure), anthropometric measurements (eg, body mass index), and routine laboratory assessments (eg, comprehensive metabolic panels) collected in clinical practice can be used to calculate a robust AL measure. These results are meaningful as most measures of AL include some variables that are not routinely collected in the clinical care of patients with breast cancer (eg, C-reactive protein),^7^ thus limiting their inclusion and use in clinical practice.

The association between social and economic marginalization and high AL is a reliable finding in the literature.^28,29^ Membership in socially and economically marginalized groups is defined as facing structural inequity and systemic inequality perpetuated by discriminatory, sexist, racist, homophobic, and classist sociocultural norms and governmental policies.^30^ Examples of groups facing historical and current social and economic marginalization in the US include Black people, women, single/unpartnered people, and individuals with low socioeconomic status (SES).^30,31,32,33^ Studies examining AL in Black people^34^ and individuals with low SES^35^ indicate these groups have higher AL than White people or those with high SES. This is consistent with our results of a higher AL in Black women and those with Medicaid insurance––a proxy for individuals with low SES. The association between elevated AL and social and economic marginalization is meaningful as it indicates AL may be a biological correlate of exposures to adverse socioenvironmental stressors, ie, structural and systemic discrimination. Additionally, the association between AL, socioeconomic marginalization (eg, black race or low SES),^8,36^ aggressive tumor characteristics (eg, estrogen receptor-negative breast cancer)^14^ and worse all-cause mortality provides a new conceptual framework to better understand socioeconomically and racially rooted disparities in breast cancer outcomes. In essence, AL provides clinicians with a means to measure the stress-related responses to socioenvironmental stressors in ways different from existing measures, such as patient-reported outcomes (eg, distress thermometer) or risk assessment tools (eg, Charlson Comorbidity Index).

### Strengths and Limitations

One strength of this study is the use of a clinical population of breast cancer patients from the electronic medical record and the cancer registry. Furthermore, the creation of an AL measure using routinely collected biomarkers demonstrates the feasibility of its use in clinical practice.

The limitations of this study include selection bias and generalizability due to using data from 1 institution. However, the sociodemographic and clinical characteristics of the study population are similar to larger population^37^ and hospital-based registries.^38^ Other limitations include that the study AL measure was limited by the availability of biomarkers data in IHIS and all biomarkers were not collected at the same time. Nonetheless, our approach to calculating AL is consistent with other breast cancer epidemiologic studies on AL.^16^

## Conclusions

In this cohort study of patients with breast cancer, elevated AL was associated with higher all-cause mortality and membership in groups facing social and economic marginalization. Future research is needed to discover the biological and behavioral mechanisms of the association between AL and mortality.

## Notes

Supplement 1. **eTable 1. **List of Postsurgical Complications **eTable 2. **Descriptive Statistics for Allostatic Load Biomarkers **eTable 3.** Crude and Adjusted Analyses Between Allostatic Load Biomarkers and Mortality **eFigure 1.** Crude (A) and Adjusted (B) Hazard Ratios (HR) of All-cause Mortality for Allostatic Load Scores Relative to 0 Allostatic Load Score **eFigure 2.** Adjusted Hazard Ratios (HR) of All-cause Mortality for Allostatic Load Scores Relative to 0 Allostatic Load Score

Supplement 2.
Data Sharing Statement
