# Supplementary material for: Association of Allostatic Load With All-Cause Mortality in Patients With Breast Cancer
Source: JAMA Netw Open. 2023 May 18;6(5):e2313989. doi: 10.1001/jamanetworkopen.2023.13989 (PMC10196875; doi:10.1001/jamanetworkopen.2023.13989)
Supplement: Supplement 1. — eTable 1. List of Postsurgical Complications eTable 2. Descriptive Statistics for Allostatic Load Biomarkers eTable 3. Crude and Adjusted Analyses Between Allostatic Load Biomarkers and Mortality eFigure 1. Crude (A) and Adjusted (B) Hazard Ratios (HR) of All-cause Mortality for Allostatic Load Scores Relative to 0 Allostatic Load Score eFigure 2. Adjusted Hazard Ratios (HR) of All-cause Mortality for Allostatic Load Scores Relative to 0 Allostatic Load Score [file jamanetwopen-e2313989-s001.pdf]

Supplemental Online Content

Obeng-Gyasi S, Elsaid MI, Lu Y, et al. Association of allostatic load with all-cause mortality in patients with breast cancer. *JAMA Netw Open*. 2023;6(5):e2313989. doi:10.1001/jamanetworkopen.2023.13989

eTable 1. List of Postsurgical Complications
eTable 2. Descriptive Statistics for Allostatic Load Biomarkers
eTable 3. Crude and Adjusted Analyses Between Allostatic Load Biomarkers and Mortality
eFigure 1. Crude (A) and Adjusted (B) Hazard Ratios (HR) of All-cause Mortality for Allostatic Load Scores Relative to 0 Allostatic Load Score
eFigure 2. Adjusted Hazard Ratios (HR) of All-cause Mortality for Allostatic Load Scores Relative to 0 Allostatic Load Score

This supplemental material has been provided by the authors to give readers additional information about their work.

© 2023 Obeng-Gyasi S et al. *JAMA Network Open.*

**eTable 1. List of Postsurgical Complications**
Sepsis
Pneumonia
Deep vein thrombosis
Acute respiratory distress syndrome
Surgical Site infection
Urinary tract infection
Acute renal failure
Acute myocardial infarction
Cardiopulmonary arrest
Stroke
Rebleeding/hematoma
Disruption of the wound
Post-surgical hematoma formation/infection
Axillary Vein injury
Nerve Injury
Seroma

© 2023 Obeng-Gyasi S et al. *JAMA Network Open.*

**eTable 2.** Descriptive Statistics for Allostatic Load Biomarkers		
**Biomarkers**	**Mean (SD)**	**Median (Q_1, Q_3)**
**Allostatic Load Score**[a]	2.6 (1.7)	2.0 (1.0, 4.0)
**Alkaline Phosphatase per U/L**	68.8 (24.4)	65.0 (52.0, 80.0)
**Albumin per g/dL**	4.3 (0.4)	4.2 (4.0, 4.4)
**Serum Creatinine per mg/dL**	0.8 (0.3)	0.78 (0.68, 0.90)
**Heart Rate**	79.6 (14.9)	78.0 (70.0, 87.0)
**White Blood Cell Count per K/uL**	7.6 (2.6)	7.1 (5.9, 8.6)
**Body Mass Index per Kg/m²**	30.4 (7.5)	29.2 (25.0, 34.5)
**Blood Pressure Diastolic per mmHg**	78.5 (11.4)	77.0 (71.0, 86.0)
**Blood Pressure Systolic per mmHg**	138.7 (18)	138.0 (127.0, 148.0)
**Blood Urea Nitrogen per mg/dL**	14.9 (5.7)	14.0 (11.0, 17.0)
**Glucose per mg/dL**	112.4 (45.2)	99 (89, 118)

[a]Allostatic load (range 0 to 10) included biomarkers for alkaline phosphatase, albumin, creatinine serum, heart rate, white blood cell count, body mass index (BMI), blood pressure diastolic, blood pressure systolic, blood urea nitrogen, and glucose.

© 2023 Obeng-Gyasi S et al. *JAMA Network Open.*

**eTable 3. Crude and Adjusted Analyses Between Allostatic Load Biomarkers and Mortality**			
**Biomarkers**	**Cutoff Units**	**Crude**	**Adjusted[a]**
		**HR (95% CI)**	**HR (95% CI)**
**High Allostatic Load**	≥ 3[†]	**1.93 (1.50 - 2.50)**	**1.51 (1.11 - 2.07)**
**Alkaline Phosphatase**	> 126 U/L	**2.27 (1.18 - 4.36)**	1.75 (0.90 - 3.39)
**Albumin**	< 3.5 g/dL	**2.98 (1.61 - 5.52)**	**1.93 (1.04 - 3.57)**
**Serum Creatinine**	>1.3 mg/dL	**2.91 (2.00 - 4.24)**	**1.67 (1.06 - 2.63)**
**Heart Rate**	>100	**1.57 (1.04 - 2.38)**	1.39 (0.91 - 2.13)
**White Blood Cell Count**	>11.19 K/uL	1.08 (0.67 - 1.75)	0.82 (0.50 - 1.34)
**Body Mass Index**	<18.5 or >25 Kg/m^2	**1.49 (1.10 - 2.03)**	1.28 (0.93 - 1.77)
**Blood Pressure Diastolic**	≥90 mmHg	1.21 (0.87 - 1.67)	1.19 (0.84 - 1.70)
**Blood Pressure Systolic**	≥130 mmHg	0.90 (0.70 - 1.16)	**0.75 (0.57 - 0.97)**
**Blood Urea Nitrogen**	>25 mg/dL	**3.32 (2.21 - 4.98)**	**2.12 (1.30 - 3.46)**
**Glucose**	≥100 mg/dL	**1.44 (1.10 - 1.88)**	0.79 (0.60 - 1.05)
[a] Adjusted for allostatic load, alkaline phosphatase, albumin, creatinine serum, heart rate, white blood cell count, body mass index (BMI), blood pressure diastolic, blood pressure systolic, blood urea nitrogen, and glucose			
[b] Allostatic load included biomarkers for alkaline phosphatase, albumin, creatinine serum, heart rate, white blood cell count, body mass index (BMI), blood pressure diastolic, blood pressure systolic, blood urea nitrogen, and glucose; High allostatic load was defined as a total allostatic load score (range 0 to 10) greater than the median			
HR=Hazard Ratio; CI=Confidence Interval			

© 2023 Obeng-Gyasi S et al. *JAMA Network Open.*

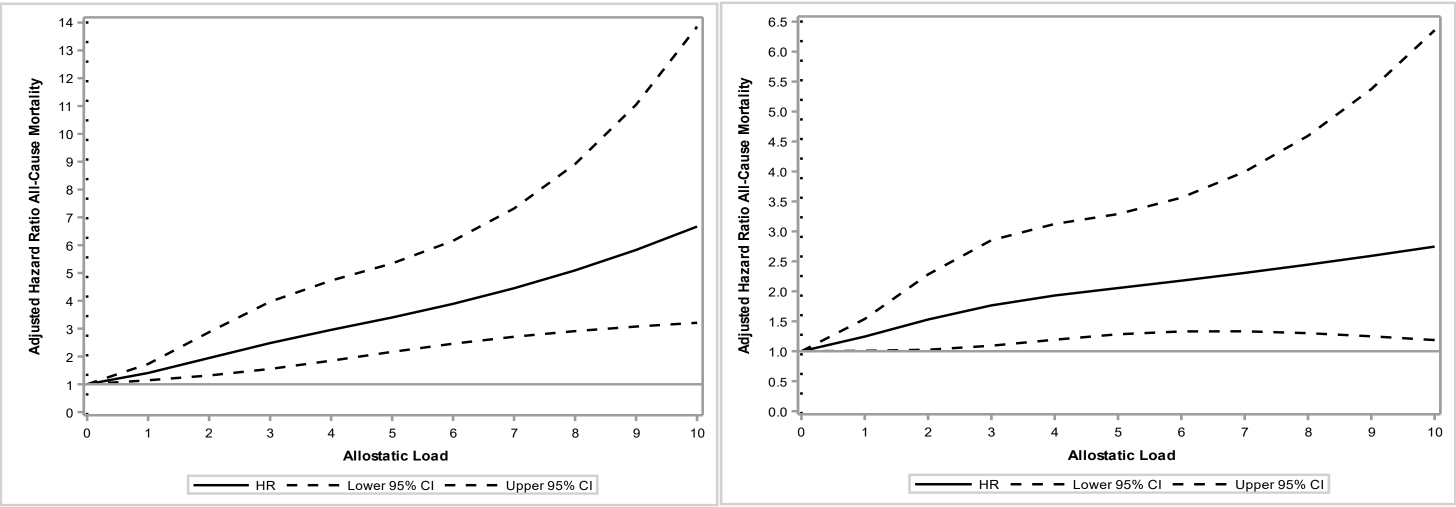

eFigure 1: Crude (A) and Adjusted* (B) Hazard Ratios (HR) of All-cause Mortality for Allostatic Load Scores Relative to 0 Allostatic Load Score (n=4,459)
* Models were adjusted for age group, race, ethnicity, health insurance, marital status, history of alcohol consumption, ever-smoker, molecular subtype, cancer stage, mastectomy, lumpectomy, surgical complications, hormone therapy, radiation therapy, chemotherapy, sentinel lymph node biopsy only, axillary lymph node biopsy only, both sentinel and axillary lymph node biopsies

© 2023 Obeng-Gyasi S et al. *JAMA Network Open.*

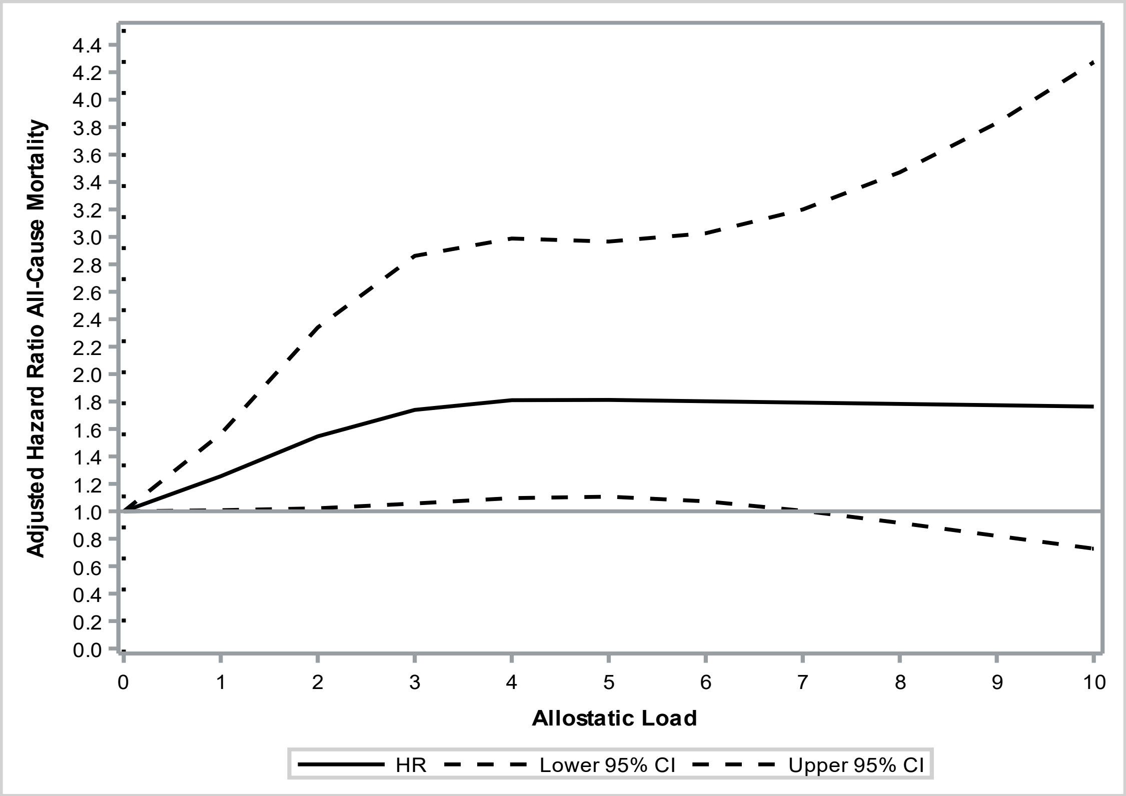

eFigure 2. Adjusted* Hazard Ratios (HR) of All-cause Mortality for Allostatic Load Scores Relative to 0 Allostatic Load Score (n=4,459)

* Models were adjusted for age group, race, ethnicity, health insurance, marital status, history of alcohol consumption, ever-smoker, molecular subtype, cancer stage, mastectomy, lumpectomy, surgical complications, hormone therapy, radiation therapy, chemotherapy, sentinel lymph node biopsy only, axillary lymph node biopsy only, both sentinel and axillary lymph node biopsies and Charlson Comorbidity Index

© 2023 Obeng-Gyasi S et al. *JAMA Network Open.*
