# Supplementary material for: Association of Allostatic Load With All-Cause Mortality in Patients With Breast Cancer
Source: JAMA Netw Open. 2023 May 18;6(5):e2313989. doi: 10.1001/jamanetworkopen.2023.13989 (PMC10196875; doi:10.1001/jamanetworkopen.2023.13989)
Supplement: Supplement 2. — Data Sharing Statement [file jamanetwopen-e2313989-s002.pdf]

Data Sharing Statement

Obeng-Gyasi. Association of Allostatic Load with All-Cause Mortality in Patients with Breast Cancer. *JAMA Netw Open*. Published May 18, 2023.
doi:10.1001/jamanetworkopen.2023.13989

Data
Data available: No

Additional Information
Explanation for why data not available: Data requests should be directed toward the Ohio State University Office of Responsible Research Practices. To gain access to the data requesters will need to sign a data access agreement with the Ohio State University.
